# Optimizing plant density and balancing NPK inputs in combination with innovative fertilizer product for sustainable maize production in North China Plain

**DOI:** 10.1038/s41598-022-13736-7

**Published:** 2022-06-18

**Authors:** Tesema Feyissa, Shuaixiang Zhao, Hailong Ma, Zhiping Duan, Weifeng Zhang

**Affiliations:** 1 Key Laboratory of Plant-Soil Interactions, College of Resources and Environmental Sciences, National Academy of Agriculture Green Development, China Agricultural University, Ministry of Education, Beijing, 100193 China; 2 Department of Crop Production, Holeta Poly Technic College, P.O.Box 11, Holeta, Ethiopia

**Keywords:** Plant sciences, Ecology, Environmental sciences

## Abstract

Excessive NPK inputs but low grain yield and high environmental impact are common issues in maize production in North China Plain (NCP). The objective of our study was to test whether a combined strategy of optimizing plant density, balancing NPK input, and innovating one-time fertilizer products could achieve a more sustainable maize production in NCP. Thus, a field experiment was conducted at Luanna County NCP with the treatments of unfertilized control (CK), farmer practice (FP, conventional plant density and NPK input), conventional one-time urea-based coated fertilizer (CF, optimized plant density and NPK input), and five newly designed innovative one-time NPK fertilizers of ammonium sulphate and urea synergy (IF, optimized plant density and NPK input), innovative fertilizer with various additives of urea inhibitors (IF + UI), double inhibitors (IF + DI), micro-organisms (IF + MI), and trace elements (IF + TE)*.* The grain yield, N sustainability indicators (N use efficiency NUE, partial factor productivity of N PFPN, and N surplus), and cost-benefits analysis were examined over the maize growing season of 2020. Results had shown that on average the five innovative fertilizers (IF, IF + UI, IF + DI, IF + MI, and IF + TE) and CF that had optimized plant density and NPK input achieved 13.5%, 98.6%, 105.9%, 37.4% higher yield, PFPN, NUE, net-benefits as well as 207.1% lower N surplus compared with FP respectively. Notably, the innovative fertilizer with various effective additives (IF + UI, IF + DI, IF + MI, and IF + TE) which can be commonly found in the fertilizer market hadn’t resulted in a significant improvement in yield and NUE rather a greater cost and lower net benefits in comparison to IF. In summary, our study highlighted the effectiveness of the combined strategy of optimized plant density, balancing NPK input, and innovative NPK fertiliser on sustainable maize production in NCP, however, the innovative fertilisers with effective additives should be properly selected for better economic benefits.

## Introduction

Sustainable crop production facing joint challenges in China^1,2^ since the yield increase here relies heavily on intensive nutrient use and generate serious environmental costs^3,4^ For example, the average nitrogen (N) application in China is over 300 kg ha^−1^ which is almost 4 times the world's 70 kg ha^−1^^5^. Less than half of N applied in China is taken up by crops with an average nitrogen use efficiency (NUE) of 25%^6^, while the rest is largely lost in the environment^7,8^ eventually leading to enhanced soil acidification^9,10^, and substantial reactive nitrogen (Nr) loss^11–13^. Hence, an urgent question arises on how to address a more sustainable crop production in China.

Globally, China is the second highest maize producer with the maize production accounting for more than one-third of country’s total cereal production^14^. However, currently, the farmer often produces maize in an unsustainable way, including overuse of N, phosphorus (P), and potassium (K), unwell selection of site-suited fertilizer products, inappropriate cultivation of plant density, and unsustainable management of soil and irrigation^15,16^. Maize production strategies toward sustainability have been studied well, including optimizing plant density, knowledge-based fertilizer practices, and efficient fertilizers^17^. Optimizing plant density generates higher yield and NUE due to the improved potential for capturing resources of water, nutrients, and solar radiations^18–20^. Knowledge-based fertilizer use approaches can be generalized by the 4R principle- right rate, right time, right source, and right place for better balancing and synchronizing nutrient delivery and crop demands^21–23^. Efficiency fertilizers includes; (i) urease inhibitor (UI) that delays the hydrolyzation rate of urea to ammonium (NH_4_^+^) to reduce ammonia (NH_3_) emission, (ii) nitrification inhibitor (NI) that blocks the microbial conversion of NH_4_^+^ to NO_3_^−^ to reduce leaching, (iii) polymer-coated material (PCF) to slow the release of nutrient for better supply to crop uptake, and (iv) soil microbes inoculations (MI) to stimulate the rhizosphere nutrient cycling and root growth^24–27^. Although the effectiveness of single strategies mentioned above has often been studied, the integrated effect of them is lacking. Understanding the integrated effect is an urgency because it is the multiple factors refer to agronomy, technology, and fertilizer products that restrict the on-farming practice, therefore demands integrated solutions.

Here, we have determined the effects of the combined strategy of optimizing plant density, balancing NPK input, and innovating one-time fertilizer products on maize yield, NUE, N surplus and economic benefit by a field experiment carried out in North China Plain (NCP). The present study aimed to evaluate whether the combined strategy (i) could obtain more yield attainable level, (ii) improve NUE, (iii) reduce N surplus, and (iv) increase net benefits.

## Materials and methods

### Description of the experimental site

**Figure 1:**
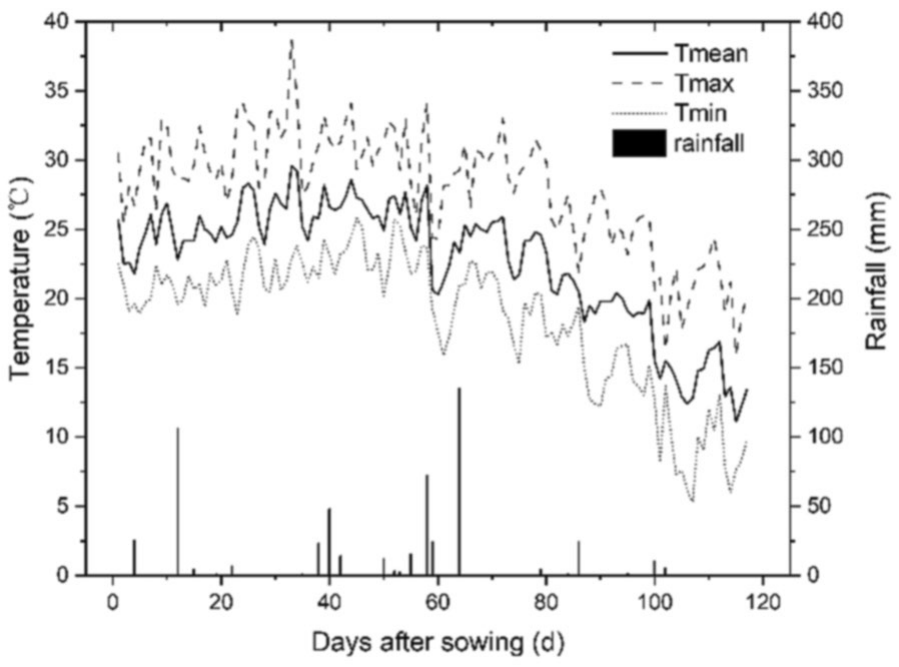
The daily mean, maximum, and minimum temperature and rainfall at Luanna County, Hebei Province, China during maize growing season June 22-October 16, 2022.

The field experiment was conducted at NCP, North-East of Hebei Province Luanna County 39°27′19″ N Latitude, 118°36′15.3″ E Longitude with an elevation of 11 masl. Luanna County is categorized as a semi-humid climate zone. The long-term (1990–2020) mean annual temperature of the area is 10.6 °C. The annual precipitation ranges from 400 to 800 mm intense heavily in summer. The field experiment was conducted in winter wheat–summer maize rotation systems of the 2020 season. The annual daily maximum, minimum, and average temperature, and rainfall during field trait (from 22nd June to 16th October in 2020) are shown here in Fig. 1. The average temperature and rainfall from planting to harvest were 22.7 °C and 4.7 mm. Rotational cropping of winter wheat/summer maize with two harvests per year is the dominant cropping system. The farmers in the study area are mainly engaged in agricultural production and have good agricultural practices and experiences.

### Innovative fertilizer and other products

In this study, we designed an innovative fertilizer product of ammonium sulfate urea (28-5-5), following the nutrient balance method to obtain the site-suited NPK formular for Luanna County NCP. The N sources varied with fertiliser products (Table 1). In farmer practice (FP), the N source was urea (46-0-0). In conventional fertilization (CF, 28-6-9), the N source was mainly large particles coated urea. In innovative fertilizer (IF, 28-5-5), the N sources were designed with mixed ammonium sulfate (20% TN) and urea (80% TN) for synergy effects^28,29^. In addition, effective additives of UI, DI, MI and TE were separately added to IF to evaluate the effective additive benefits.

**Table 1:** Detail of treatments and nutrient inputs.

Treatment	Plant density (plant ha^−1^)	N forms (%)	Base fertilization (Kg ha^−1^)			Topdressing (Kg ha^−1^)			Total		
			N	P_2_O_5_	K_2_O	N	P_2_O_5_	K_2_O	N	P_2_O_5_	K_2_O
CK	78,000	–	–	–	–	–	–	–	–	–	–
FP	66,666	100 urea	105	110	120	245	–	–	350	110	120
CF	78,000	100 coated urea	200	42.8	64.3	–	–	–	200	42.8	64.3
IF	78,000	80 urea, 20 (NH_4_)_2_SO_4_	200	35.6	35.6	–	–	–	200	35.6	35.6
IF + UI	78,000	80 urea, 20 (NH_4_)_2_SO_4_	200	35.6	35.6	–	–	–	200	35.6	35.6
IF + DI	78,000	80 urea, 20 (NH_4_)_2_SO_4_	200	35.6	35.6	–	–	–	200	35.6	35.6
IF + MB	78,000	80 urea, 20 (NH_4_)_2_SO_4_	200	35.6	35.6	–	–	–	200	35.6	35.6
IF + TE	78,000	80 urea, 20 (NH_4_)_2_SO_4_	200	35.6	35.6	–	–	–	200	35.6	35.6

### Experimental treatment details

To estimate the maize yield potential in Luanna County, the Hybrid Maize model (Hybrid Maize, 2013) and 10 years local weather data were used, indicating maize potential yield of about 15.6 Mg ha^−1^ at 14% moisture content when the density is about 78,000 plants population ha^−1^. The field experiment was laid out as a randomized complete block design (RCBD) with three replicates. The experiment had 8 treatments. Thus, there are 8 × 3 = 24 plots. The plant design was 66,666 plants ha^−1^ for farmer practice (FP) while the others’ plant density was 78,000 plants ha^−1^. Each plot area was 40 (8 × 5) m^2^, with the total area of 960 m^2^ excluding the area between plots. Each plot had nine rows of which three to seven central rows were used for data collection and analysis and two rows of each plot side were left as border for the whole treatments except FP which had eight rows. The two outermost rows from each plot and one plant from both ends of each row were considered as a border. The following was the table detail of treatments and nutrient inputs (Table 1).

### Experimental field management

The maize (*Zea mays *L.) variety of Deng-Hai (605) from the local area was selected on June 22. Growing land was plowed once by tractor before sowing. Two seeds per hole were sown by dibbling at about 5 cm depth and the seeds were covered with soil manually to ensure adequate emergence. Thinning was done 10 days after emergence to one plant per hole to maintain the specified intra plant spacing. Base fertilization was applied at the base by hand having a 5–8 cm distance from the seeds to avoid the toxicity of fertilizer. For FP 30% of the N fertilizer was applied as base fertilizer while 70% was applied as top-dressing at thirty-five days after sowing. During the maize growing season and critical stages at emergence and silking, a little irrigation was used to avoid water stress and achieve a high yield. To avoid a lodging growth inhibitor was applied once at V8 growth stages. One time herbicide was applied to control the weeds. Finally, the experiment was harvested on October 16 having 122 growing degree days to allow the late cobs filling and enough time for maturity.

### Soil sampling and analysis

Soil samples were taken in a zigzag pattern before planting randomly from the experimental site at depth of 0–20 cm across the experimental field from 20 spots using an auger before planting and were composited. About 1.0 kg of soil composite sample was taken using a polythene bag to soil laboratory test. Furthermore, after harvest 500 g composite samples from three randomly selected spots diagonally per plot were taken to test the soil nutrient content by using an auger at 0–20 cm. The sample was dried at room temperature, systematically mixed, and crushed to pass through a 2 mm sieve in preparation for laboratory analysis. The sample was analyzed for soil texture, pH, organic carbon (SOC), total nitrogen (TN), available nitrogen (Ava. N), available phosphorus (Ava. P), and available potassium (Ava. K) following standard analytical procedures.

### Maize and biomass yield measurement

#### Yield and yield components measurement method

After the corn fills and matured (2020-10-17) the yield was measured by designing twenty (20) square meters in the middle of each plot as the production area. The number of cobs per plant at harvest was counted from the production area. The total weights of cobs were measured and divided to get the mean cobs weights to select ten representative cobs for all yield and yield component evaluation and analysis.

#### Aboveground dry biomass yield (Mg ha^−1^)

It was recorded by taking the weight of 5 randomly selected representative plants at physiological maturity by using sensitive balance. The sample biomass was cut from the ground fresh plants into kraft paper bags, put in a constant temperature drying oven at 105 °C for 30 min to deactivate enzymes, and then oven-dried at 80 °C for 72 h to determine dry matter yield. The average dry biomass per plant was multiplied by the number of total plants in the net plot area at harvest. It was expressed as dry biomass in Mg ha^−1^. Furthermore, this biomass yield was used for the calculation of the harvest index.

#### The number of ears ha^−1^

During harvesting, the number of ears from the production area was counted and scaled up to a hectare basis.

#### Grain yield (Mg ha^−1^)

It was recorded by threshing ten representative ears per plot by using field balance and converted to the total area per plot. The grain yield was cleaned and converted into Mg ha^−1^, the yield was adjusted to a 14% moisture level, and finally, scaled up to a hectare basis.

#### Hundred seed weight (g)

It was recorded by taking the weight of 100 randomly sampled seeds from the grain yield per plot by using a sensitive balance and the weight was adjusted to a 14% moisture level. The grain moisture content was measured by using a grain moisture meter while the kernel was counted by using a kernel counter.

#### Harvest index (HI)

It was computed as the ratio of grain yield (kg ha^−1^) to total above-ground dry biomass per ha.

### Nitrogen sustainability index analysis

#### Partial factor productivity of nitrogen (PFPN)

Partial factor productivity of nitrogen fertilizer was calculated as yield per unit inputs of nitrogen fertilizer for all treatments except for CK unfertilized control (treatment with no fertilization).

#### N uptake (kg ha^−1^)

The nitrogen uptake at harvest was calculated as the formula:

$${\text{Straw N uptake }} = {\text{ SNCT }}\left( {\text{the straw N concentration}} \right) \, \times {\text{ DMSW }}\left( {\text{the dry matter of straw weights}} \right)$$

$${\text{Grain N uptake }} = {\text{ GNCT }}\left( {\text{the grains nitrogen concentration}} \right) \, \times {\text{ GW }}\left( {\text{the weight of the grains}} \right)$$

$${\text{Total N uptake }} = {\text{ straw N uptake }} + {\text{ grain N uptake}}$$

#### NUE (%)

The NUE was calculated by dividing the above-ground nitrogen uptake to applied nitrogen from fertilizer by using the following equation:

$${\text{NUE }} = \, \left( {{\text{N}}_{{\text{uptake fertilized}}} {-}{\text{ N}}_{{\text{uptake unfertilized}}} } \right)/{\text{N applied}}$$

#### N surplus (Kg N ha^−1^ yr^−1^)

The N surplus was calculated as the total of N inputs (fertilizer, irrigation, biologically fixed N, and N deposition) minus N outputs (the N removed within harvested maize products, N _yield_).

#### Cost benefits analysis

Production cost composition includes land preparation and sowing, seed, fertilizer, topdressing, irrigation, thinning, and chemical controls for herbicides and pesticides. The labor cost during maize sowing and harvesting was 15 RMB hour^−1^. Harvest output = yield × yield price (2.5 RMB kg^−1^). Lastly revenue (Net benefits) = output − production cost − labor cost.

### Statistical data analysis

All the measured parameters were subjected to analysis of variance (ANOVA) appropriate to the factorial experiment in RCBD according to IBM SPSS statistics version 25. The mean record was equated using the Duncan Least Significant Differences (LSD) test at a 5% level of significance.

### Maize collection guidelines statement

This is to confirm that all local, national or international guidelines and legislation were adhered to in the production of this study.

## Results

### Soil physical and chemical properties of the study area

The initial soil physicochemical properties (Table 2) reveal that the texture classification was sandy loam, soil pH in water was 5.62 found in the medium acids^30^, very low SOC (0.62%), and moderate Ava. P (39.8 ppm) ^31^.

**Table 2:** Physicochemical characteristics of the soil before sowing.

Indicators	pH in H_2_O	Sand	Clay	Silt	SOC	TN	Ava. N	Ava. P	Ava. K
Units		%	%	%	%	%	ppm	ppm	ppm
Value	5.62	59.8	15.3	24.9	0.62	0.08	79.2	39.8	51.2

The chemical properties of the soil after harvest (Table 3) confirmed that IF and IF + MI were significantly higher than FP with soil pH. Except for soil pH, other soil chemical properties (SOM, SOC, TN, Ava. N, Ava. P, and Ava. K) had no significant differences.

**Table 3:** The chemical characteristics of the soil after harvest.

Treatment	pH in H_2_O	SOM	SOC	TN	Ava. N	Ava. P	Ava. K
		%	%	%	ppm	ppm	ppm
CK	5.9 ± 0.3^ab^	1.1 ± 0.1^a^	0.7 ± 0.1^a^	0.07 ± 0.01^a^	63.2 ± 12.3^a^	25.9 ± 9.5^a^	51.0 ± .8.2^a^
FP	5.6 ± 0.2^b^	1.2 ± 0.2^a^	0.7 ± 0.1^a^	0.07 ± 0.01^a^	83.1 ± 15.4^a^	26.4 ± 18.5^a^	57.0 ± 4.0^a^
CF	5.8 ± 0.2^ab^	1.2 ± 0.1^a^	0.7 ± 0.1^a^	0.08 ± 0.01^a^	72.5 ± 3.2^a^	32.1 ± 8.3^a^	60.7 ± 10.0^a^
IF	6.2 ± 0.4^a^	1.4 ± 0.1^a^	0.8 ± 0.1^a^	0.08 ± 0.01^a^	71.1 ± 0.6^a^	25.3 ± 9.4^a^	54.7 ± 6.6^a^
IF + UI	6.0 ± 0.2^ab^	1.1 ± 0.3^a^	0.7 ± 0.1^a^	0.08 ± 0.01^a^	64.0 ± 5.9^a^	29.2 ± 2.1^a^	60.3 ± 5.5^a^
IF + DI	5.9 ± 0.1^ab^	1.2 ± 0.1^a^	0.7 ± 0.1^a^	0.08 ± 0.01^a^	67.2 ± 12.3^a^	34.1 ± 7.5^a^	54.7 ± 5.0^a^
IF + MI	6.2 ± 0.2^a^	1.4 ± 0.1^a^	0.8 ± 0.1^a^	0.09 ± 0.01^a^	81.3 ± 7.4^a^	31.4 ± 5.1^a^	56.0 ± 4.0^a^
IF + TE	5.9 ± 0.1^ab^	1.4 ± 0.2^a^	0.8 ± 0.1^a^	0.09 ± 0.01^a^	76.4 ± 6.2^a^	28.7 ± 2.2^a^	64.5 ± 7.8^a^

*CK* unfertilized control, *FP* farmer practice, *CF* conventional fertilization, *IF* innovative fertilizer, *IF* + *UI* innovative fertilizer and urea inhibitors additives, *IF* + *DI* innovative fertilizer and double inhibitors additives, *IF* + *MI* innovative fertilizer and micro-organisms additives, and innovative fertilizer and micro-organisms trace elements additives (IF + TE). All values are reported as mean ± SD, n = 3. The values followed by the different letters show statistically significant differences at *P* < 0.05.

### Maize biomass, crop yields, and harvest index

#### Above-ground dry biomass

The CK had the lowest mean stalk, grain, and total dry matter while the highest was obtained from innovative fertilizers (Table 4). In all types of dry matter, innovative fertilizer treatments (IF, IF + UI, IF + DI, IF + MI, & IF + TE) were significant to CK. On average, the five innovative fertilizer total dry matter (IF, IF + UI, IF + DI, IF + MI, & IF + TE) were 12% and 20.5% higher than FP and CK respectively. From innovative fertilizers, IF and IF + DI had achieved the highest (22.8 Mg ha^−1^) and lowest (22.0 Mg ha^−1^) total dry matter though the result was insignificant.

**Table 4:** Mean values of above-ground dry biomass yield (Mg ha^−1^) and harvest index (%).

Treatment	Stalk dry matter (Mg ha^−1^)	Grain dry matter (Mg ha^−1^)	Total dry matter (Mg ha^−1^)	Harvest index (%)
CK	9.7 ± 0.8^c^	8.7 ± 0.9^c^	18.4 ± 0.8^c^	46.1 ± 2.3^a^
FP	11.0 ± 1.2^ab^	8.7 ± 0.5^c^	19.8 ± 0.5^bc^	45.0 ± 1.7^a^
CF	11.5 ± 1.0^ab^	9.5 ± 0.3^b^	20.9 ± 1.1^ab^	46.7 ± 2.4^a^
IF	12.5 ± 1.8^a^	10.3 ± 0.7^a^	22.8 ± 0.7^a^	46.4 ± 4.0^a^
IF + UI	11.7 ± 1.7^ab^	9.8 ± 0.2^ab^	21.5 ± 1.9^ab^	46.2 ± 3.4^a^
IF + DI	12.2 ± 2.1^ab^	9.8 ± 0.6^ab^	22.0 ± 1.6^ab^	45.9 ± 5.5^a^
IF + MI	12.4 ± 0.5^a^	9.7 ± 0.1^ab^	22.1 ± 0.6^ab^	44.9 ± 0.3^a^
IF + TE	12.0 ± 0.7^ab^	10.4 ± 0.1^a^	22.5 ± 0.6^a^	47.1 ± 2.2^a^

*CK* unfertilized control, *FP* farmer practice, *CF* conventional fertilization, *IF* innovative fertilizer, *IF* + *UI* innovative fertilizer and urea inhibitors additives, *IF* + *DI* innovative fertilizer and double inhibitors additives, *IF* + *MI* innovative fertilizer and micro-organisms additives, and innovative fertilizer and micro-organisms trace elements additives (IF + TE). All values are reported as mean ± SD, n = 3. The values followed by the different letters show statistically significant differences at *P* < 0.05.

#### Harvest index

The mean harvest index ranges from 44.9 to 47.1% (Table 4) however the result was insignificant.

#### The number of ears

The IF + UI and FP achieved the highest (76,667) and lowest (76,111) the number of ear ha^−1^ respectively (Table 5). Statistically, all  innovative fertilizers (IF, IF + UI, IF + DI, IF + MI, & IF + TE), CF, and CK were significant to FP. From innovative fertilizer, IF + UI and IF + DI achieved the highest (76,667) and lowest 75,889 number of ears ha^−1^ respectively however the result was insignificant.

**Table 5:** Mean number of ear ha^−1^, number of kernels ear^−1^, 100 kernel weights, grain yield.

Treatment	Number of ears ha^−1^	Number of kernel ear^−1^	100 kernel weights (g)	Grain yield (Mg ha^−1^)
CK	76,111 ± 962^a^	539 ± 51^c^	26.2 ± 1.5^a^	10.1 ± 0.1^c^
FP	63,889 ± 962^b^	623 ± 70^ab^	28.1 ± 0.5^a^	10.2 ± 0.6^c^
CF	75,444 ± 1425^a^	596 ± 87^ab^	26.3 ± 1.4^a^	11.0 ± 0.3^b^
IF	76,111 ± 1546^a^	633 ± 68^a^	27.1 ± 0.7^a^	12.0 ± 0.3^a^
IF + UI	76,667 ± 1167^a^	594 ± 61^ab^	27.0 ± 0.9^a^	11.4 ± 0.8^ab^
IF + DI	75,889 ± 1213^a^	599 ± 57^ab^	27.4 ± 1.3^a^	11.4 ± 0.7^ab^
IF + MI	76,112 ± 705^a^	602 ± 65.0^ab^	27.1 ± 1.2^a^	11.3 ± 0.2^ab^
IF + TE	76,222 ± 907^a^	639 ± 69^a^	27.2 ± 1.1^a^	12.1 ± 0.1^a^

*CK* unfertilized control, *FP* farmer practice, *CF* conventional fertilization, *IF* innovative fertilizer, *IF* + *UI* innovative fertilizer and urea inhibitors additives, *IF* + *DI* innovative fertilizer and double inhibitors additives, *IF* + *MI* innovative fertilizer and micro-organisms additives, and innovative fertilizer and micro-organisms trace elements additives (IF + TE). All values are reported as mean ± SD, n = 3. The values followed by the different letters show statistically significant differences at *P* < 0.05.

#### The number of kernels per ear

The IF + TE and CK ensured the highest (639) and lowest (539) number of kernels respectively (Table 5). All fertilization (FP, CF, IF, IF + UI, IF + DI, IF + MI, and IF + TE) treatments were statistically significant to CK. From innovative fertilizer, IF + TE and IF + UI achieved the highest (639) and lowest (594) number of kernels per ear respectively though the result was insignificant.

#### Hundred kernel weight (g)

The FP achieved the highest (28.1) while CK had the lowest (26.2) hundred kernels weight respectively however not statistically significant (Table 5).

#### Grain yield

The highest (12.1 Mg ha^−1^) and lowest (10.1 Mg ha^−1^) grain yield was found from IF + TE and CK respectively (Table 5). As a result, five innovative fertilizers (IF, IF + UI, IF + DI, IF + MI, and IF + TE) and CF were significant to FP and CK. On average the five innovative fertilizers (IF, IF + UI, IF + DI, IF + MI, and IF + TE) had achieved 14.9% and 13.8% yields higher than CK and FP respectively. Additionally, IF + TE and IF were significantly higher than CF. From innovative fertilizer, IF + TE and IF + UI attained the highest (12.1 Mg ha^−1^) and lowest (11.3 Mg ha^−1^) grain yield respectively though the result was insignificant.

### Nitrogen sustainability index analyses

#### N uptake

**Figure 2:**
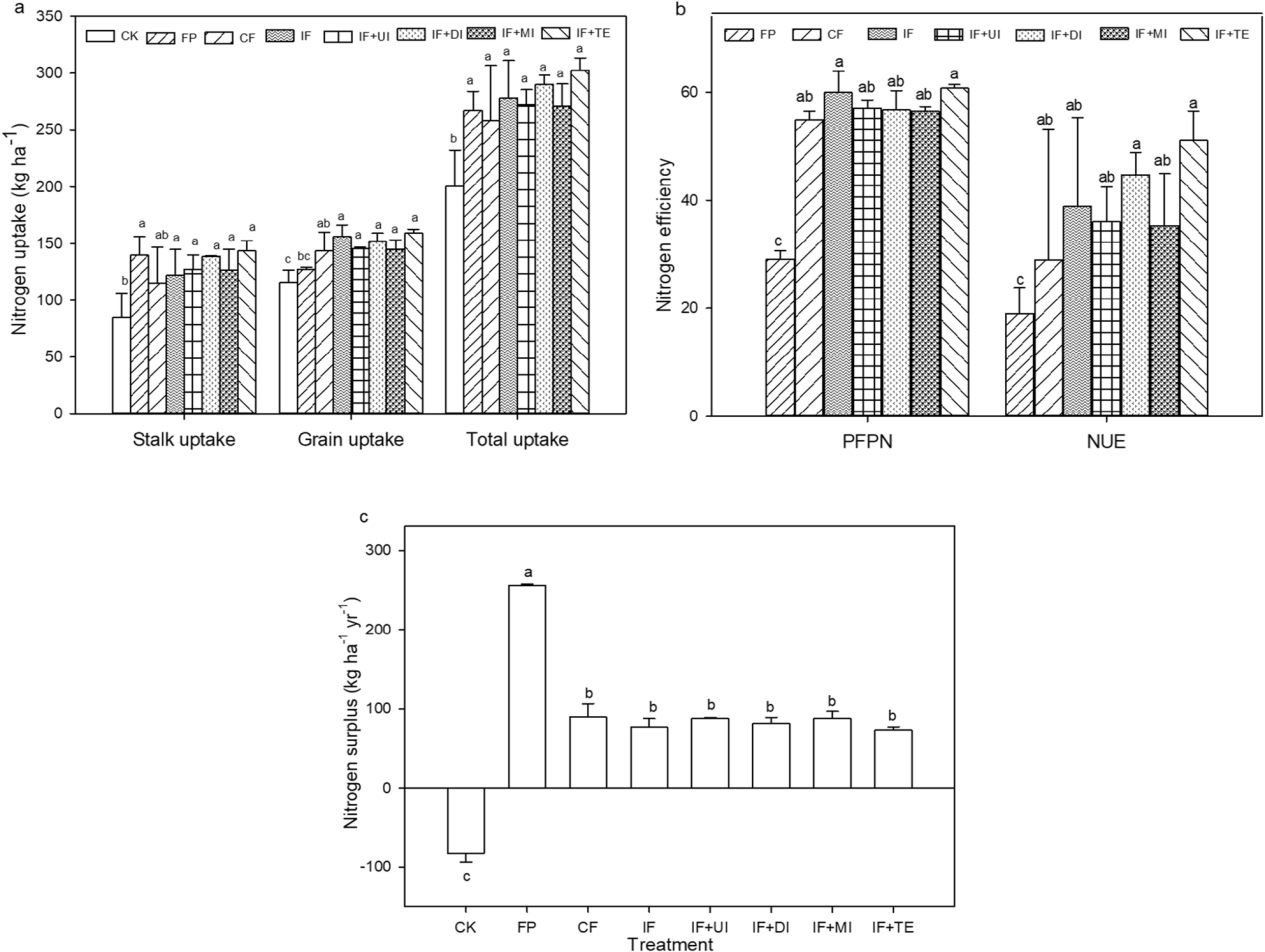
Nitrogen uptake (**a**), Partial factor productivity of nitrogen (PFPN) (**b**), Nitrogen use efficiency (NUE) (**b**), and Nitrogen surplus (total N input minus total N uptake) (**c**). CK, unfertilized control; FP, farmer practice; CF, conventional fertilization; IF, innovative fertilizer; IF + UI, innovative fertilizer and urea inhibitors additives; IF + DI, innovative fertilizer and double inhibitors additives; IF + MI, innovative fertilizer and micro-organisms additives, and innovative fertilizer and micro-organisms trace elements additives (IF + TE). All values are reported as mean ± SD, n = 3. The values followed by the different letters show statistically significant differences at *P* < 0.05.

In all N uptake forms, CK had always the lowest N uptake while the highest N uptake was found from innovative fertilizers irrespective of N forms (Fig. 2a). All innovative fertilizers (IF, IF + UI, IF + DI, IF + MI, & IF + TE), CF, and FP had achieved statistically similar stalk N uptake ranging from 115.0 to 143.5 kg N ha^−1^. All innovative treatments had higher grain N uptake than CF, FP, and CK. From innovative fertilizer IF + TE and IF + MI had achieved the highest (302.6%) and lowest (271.2%) total N uptake however the result was insignificant. Furthermore, the total N uptakes in fertilization treatment were statistically similar but substantially significantly higher than the CK.

#### PFPN

The FP (29.0 kg yield/kg N) was half lower than the PFPN of other treatments (55.0–60.7 kg yield/kg N) (Fig. 2b). Statistically, five innovative fertilizers (IF, IF + UI, IF + DI, IF + MI, & IF + TE) and CF were significantly higher than FP. From innovative fertilizer, IF + TE and IF + MI attained the highest (60.7 kg yield/kg N) and lowest (56.5 kg yield/kg N) PFPN respectively although the result was insignificant.

#### NUE

The NUE ranges from 19.0 to 51.1% while the highest and lowest NUE was found from IF + TE and FP respectively (Fg. 2b). All innovative fertilizers (IF, IF+UI, IF+DI, IF+MI, & IF+TE) and CF were significantly higher than FP. From innovative fertilizer, IF + TE and IF + MI had achieved the highest (51.1%) and lowest (35.4%) NUE however the result was insignificant.

#### N surplus

The FP achieved the significant and highest N surplus (255.5 kg ha^−1^) more than two times higher than innovative fertilizers which range from (73.9–88.7 kg ha^−1^) (Fig. 2c). The result was insignificant among innovative fertilizers. On the other hand, CK had achieved the lowest and negative N surplus (− 82.5 kg ha^−1^).

### Costs benefits

The fertilizer cost of the innovative fertilizer (1395–1530 RMB ha^−1^) was significantly lower than FP (3600 RMB ha^−1^) and CF (2175 RMB ha^−1^) (Table 6). The FP and CK had the highest and lowest cost respectively. The IF + TE and CK had the highest (30,250 RMB ha^−1^) and lowest (25,250 RMB ha^−1^) output respectively. The net benefits indicated that IF + TE and FP had achieved the highest (21,250 RMB ha^−1^) and lowest (14,250 RMB ha^−1^) net benefits. The net income performance of the treatment in the sequence was: IF + TE, IF, IF + UI, IF + DI, IF + MI, CF, CK, and FP.

**Table 6:** The cost and benefit analysis of different treatments (RMB ha^−1^).

Treatment	Cost structure								Total cost	Output	Net benefits	Relative to CK (%)	Relative to FP (%)
	Land preparing	Seed	Fertilizer	Irrigation	Topdressing	Thinning	Chemical control	Harvest					
CK	1800	825	0	1800	0	900	675	1500	7500	25,250	17,750	–	–
FP	1800	825	3600	1800	150	900	675	1500	11,250	25,500	14,250	− 19.7	–
CF	1800	825	2175	1800	0	900	675	1500	9675	27,500	17,825	0.4	25.1
IF	1800	825	1395	1800	0	900	675	1500	8895	30,000	21,105	18.9	48.1
IF + UI	1800	825	1470	1800	0	900	675	1500	8970	28,500	18,530	10.0	37.1
IF + DI	1800	825	1485	1800	0	900	675	1500	8985	28,500	19,515	9.9	36.9
IF + MI	1800	825	1530	1800	0	900	675	1500	9030	28,250	19,220	8.3	34.9
IF + TE	1800	825	1500	1800	0	900	675	1500	9000	30,250	21,250	19.7	49.1

*CK* unfertilized control, *FP* farmer practice, *CF* conventional fertilization, *IF* innovative fertilizer, *IF* + *UI* innovative fertilizer and urea inhibitors additives, *IF* + *DI* innovative fertilizer and double inhibitors additives, *IF* + *MI* innovative fertilizer and micro-organisms additives, and innovative fertilizer and micro-organisms trace elements additives (IF + TE). Total Cost = Land Preparation and Sowing + Seed + Fertilizer + Irrigation + Top Dressing + Thinning + Chemical Control + Harvest; Output = Yield (kg ha^-1^) X 2.5 RMB; Net Benefits = Output-Total Cost.

## Discussion

### Maize soil status

The acidity and low SOC in studied soil are mainly caused by high N inputs (over 300 kg ha^−1^) and unsustainable soil management (i.e., intensive tillage, low inputs of organic fertilizer) which are mostly found in the North China Plain^32,33^. Additionally, the texture class of the present maize soil is sandy loam which tends to result in a low buffering capacity and a high rate of water percolation and infiltration thereby high risks of N leaching (Table 2). In the present study, we designed the innovative fertilizer with multiple N forms (NH_4_^+^-N, Urea-N) and efficiency additives (UI, DI, MI, and TE) combined with the reasonable N, P_2_O_5_, and K_2_O fertilizer and optimum plant density to explore the potential benefits on the soil. According to our result, the innovative fertilizer of IF and IF + MI soil pH (6.2) after harvest was significantly higher than FP (5.6) probably because of excess N, P_2_O_5_, and K_2_O inputs in the case of FP. It suggests that an appropriate input of nutrients is beneficial for improvement on acidic soil. However, other soil chemicals' properties (SOM, OC, TN, Ava. N, Ava. P, and Ava. K) were insignificant probably due to the experiment being conducted for one season (Table 3). Therefore, it needs further research to explore the long-term effects of innovative fertilizer on soil properties.

### Effect of innovative fertilizer on yield and yield potential

The production target is to gain a substantial and sustainable yield in unit mass which has been highlighted by numerous authors^21,34^. In this study, the five innovative fertilizers (IF, IF + UI, IF + DI, IF + MI, and IF + TE) and CF with 43% lower N input had achieved a 13.1% higher yield than FP (Table 5) which was mainly attributed to a higher plant density of 78,000 plants ha^−1^ in five innovative fertilizers and CF than 66,666 plants ha^−1^ FP (Table 1). Further results of yield components had shown that the increased grain yield per unit area is because of improved optimum plant population (number of ear ha^−1^) rather than increased grain yield per plant, with the same number of kernels ear^−1^, and 100 kernels weight.

Despite the largest N inputs from FP (350 kg ha^−1^), there was a statistically similar yield between FP and CK. Overuse of N fertilizer has adverse effects on crops by minimizing N use efficiency (NUE) and increasing nitrate leaching losses as well as contamination of groundwater^35,36^. Furthermore, long-term application of ammonia-based N fertilization like urea increases soil acidity which adversely affects soil fertility where crops fail to respond with more application of N fertilizers^37^. To know optimum N inputs levels, it is essential to know the level to which N fertilization rate is reliable with crop N needs to exploit resource utilization and sustain relatively high grain yields. In the present study, the highest grain yield was achieved under the innovative fertilizer of IF + TE (Table 5) with reasonable N inputs of 200 kg ha^−1^ (Table 1). For the five innovative fertilizers with effective additives, the result was insignificant because the soil and climate conditions where the effective additives are used may highly affect the final effects. For instance, urease inhibitors (IU) have little effect in acidic soils while the present study soil was medium acidic. Microorganisms (MI) additives have better performance on high SOC^21,38^ whereas our current study soil was low in SOC. Therefore, the effective additives should be targeted use according to the field biophysical settings.

The Hybrid Maize model estimates the yield potential of Luannan County as 15.6 Mg ha^−1^ at 14% moisture content and 14.6 Mg ha^−1^ grain dry matter when the density is about 78,000 maize plants population ha^−1^. The five innovative fertilizers (IF, IF + UI, IF + DI, IF + MI, and IF + TE) achieved about 76.9%, 73.1%, 73.1%, 72.4%, and 77.6% of yield potential while CF, FP, and CK attained 70.5%, 65.4%, and 64.7% respectively. On average, the five innovative fertilizers (IF, IF + UI, IF + DI, IF + MI, and IF + TE) achieved 74.6% yield potential while the research target was 85% yield potential of about 13.3 Mg ha^−1^. In the present finding, the experiment had not achieved the research target probably because the experiment study soil was medium acidic soil. In the medium acidic soil pH category nutrients such as phosphorus, potassium, calcium, and molybdenum are adversely affected leading to a reduction in crop yields^30^.

### Effect of innovative fertilizer on nutrient use efficiency

PFPN is an important indicator reflecting the efficiency of N fertilizer utilization. In this result, five innovative fertilizers (IF, IF + UI, IF + DI, IF + MI, and IF + TE) and CF PFPN were significantly higher than FP probably due to a reasonable amount of N inputs, optimum plant density, and high yield achieved (Fig. 2b). The five innovative fertilizers (IF, IF + UI, IF + DI, IF + MI, and IF + TE) had realized 100.5% and 5.9% higher than FP and CF respectively while IF + TE performed the highest PFPN. In this result, the high PFPN indicates that more output was produced from fertilizer application of 200 kg ha^−1^ N for innovative fertilizers than 350 kg ha^−1^ for FP^39,40^. Similar results were reported by Amanullah 2016; Draman and Almas 2009; Yan et al. 2016^41–43^ in which optimum plant density and N inputs lead to better PFPN.

Improving NUE is one of the most effective methods of increasing crop productivity while decreasing environmental degradation^44,45^. In the present study on average, the five innovative fertilizers (IF, IF + UI, IF + DI, IF + MI, & IF + TE) had achieved 41.2% NUE (Fig. 2b). The average change of five innovative fertilizers in comparison to FP and CF was 117% and 42.6% higher respectively indicating a high improvement in nitrogen use efficiency in comparison to the current farmer practice. The NUE five innovative fertilizers in descending order were IF + TE, IF + DI, IF, IF + UI, and IF + MI respectively. However, the average NUE of five innovative fertilizers (41.2%) is still below the suggested range for NUE (50−90%) according to the EU Nitrogen Expert Panel^46^ which needs further improvement.

Within farming systems, many indicators (N input, NUE indices, Soil mineral N, and N surplus) have been used to estimate potential N losses to the environment. Among these N surplus is an easily calculated indicator of the balance of N input minus N output. It has been used as a guideline for improving and promoting sustainable nutrient management within specified boundaries^47–51^. Zhang et al. 2019^46^ identified the N surplus benchmark of 40–100 kg ha^−1^ yr^−1^ N and 110−190 kg ha^−1^ yr^−1^ N for single and double-cropping systems in China respectively. In the present study, on average the five innovative fertilizers (IF, IF + UI, IF + DI, IF + MI, & IF + TE) had achieved 81.7 kg ha^−1^ yr^−1^ N for single cropping which is within the recommended range and sustainable for N management (Fig. 2c). Additionally, Zhang et al. 2019^46^ suggested a minimum productivity level (N harvest = 80 kg N ha^−1^ yr^−1^) which is almost similar to the present study. On the other hand, CK and FP had shown − 82.5 kg ha^−1^ yr^−1^ N and 255.5 kg ha^−1^ yr^−1^ N respectively for single cropping systems. Both largely positive and negative N surplus is unsustainable as the former often causes soil depletion, especially in long-term fertilization while the latter often causes high environmental risks of N losses.

### Effect of innovative fertilizer on economic analysis

From an economic perspective, the essential parameters during the assessment of agricultural systems are achieved yield and its technological parameters^52^. Farmers consider the economic benefits and risks based on the fertilizer and grain prices when newly designed fertilizer is used^9^. In the present study, in comparison to FP and CF, on average, the five innovative fertilizers (IF, IF + UI, IF + DI, IF + MI, and IF + TE) reduced the cost of production by 25.0% and 7.9% and additionally generated 11.6% and 4.8% higher output, thereby achieved 29.2% and 11.4% net benefits (Table 6). It suggests that the innovative fertilizer has more economic advantages than FP and CF because of the advantage in increased grain yield, decreased cost of fertilizer, and decreased labor costs with a one-time application. In comparison to IF only, the four innovative fertilizers with effective additives (UI, DI, MI, and TE) had an averagely increased 7.3% higher cost of production, while generated 3.9% lower output and resulted in 6.2% lower net benefits. It suggests that most innovative fertilizers with effective additives just increased the cost of production rather than obtaining more output benefits due to the limited improvement in yield.

### Potential contribution to bioeconomy of sustainability

The use of maize as a feedstock to produce bioenegy (i.e., bioethanol) could contribue to boost the bioeconomy: an innovation idea recently proposed to deliver the UN Sustainable Development Goals. However, bioenegy from maize may result in shortage of feed and food, espesically in China and Africa where the feed and food use accounted for ~ 80% of total maize consumption^53^. Our study had shown that compared with FP the five innovative fertilizers that had averagely increased maize yield and biomass by 13.4% and 12.0% repectively (Table 5). It suggests that the innovative fertilizers can be a technical approach to alleviate the “food versus fuel” debate for a better bioeconomy through the large improvements on the maize productivity in terms of grain and biomass^54^. Additionally, the substantially reduced use of mineral fertilizer (43% lower N, 68% lower P_2_O_5,_ and 70% lower K_2_O inputs) with innovative fertilizers also contributed to largely reduced use of fossil-based energy related to mineral fertilizer processing, which is the major objective of bioeconomy to replace the fossil resources^55^. Moreover, compared to MF, the five innovative fertilizers also reduced the cost of production by 25.0% and achieved 29.2% net benefits (Table 6), indicating that the innovative fertilizers has the possible market effects on regulating maize price that usually increased due to the strong demand of bioenergy use^56^. More complete and deep analyses related to life cycling assessments and/or socio-economic framework are required to determine these potential benefits on bioeconomy of sustainability.

## Conclusion

Sustainable maize production requires a high yield with less inputs as well as minor environmental impacts. Our result had demonstrated that the combined strategy of optimizing plant density, balancing NPK inputs, and innovating NPK fertilizer products is critical for sustainable maize production in NCP with more comprehensive benefits on yield, NUE, N surplus, and economic gains compared to the most common farmer practice. Meaningfully, this combined strategy is an agronomically robust and relatively easy way to adopt but much relies on innovative operations with knowledge researchers, extension governments, and fertilizer-product markets. Moreover, this combined strategy should be variable across different regionals soil and climate which may require differential plant density, nutrient inputs, and fertilizer characteristics as well as effective additives. Therefore, it highlights a regional environment-oriented design on the combined strategy to meet multiple objectives such as achieving high-cost savings, efficiency increasing as well as sustainable and green development.

## Data Availability

The data that support the findings of this study are available from Tesema Feyissa but restrictions apply to the availability of these data, which were used under license for the current study, and so are not publicly available. Data are however available from the authors upon reasonable request and with permission of Tesema Feyissa (email: tesemafayyisaa@gmail.com). {Only to be included in the email for non-research articles e.g. study protocols or literature reviews}.

## Abbreviations

NCP: North China Plain
CK: Control treatment
FP: Farmer practice
CF: Conventional fertilizer
IF: Innovative fertilizer
TE: Trace elements
UI: Urea inhibitors
DI: Double inhibitors
MI: Micro-organism’s
PFPN: Partial factor productivity of nitrogen
NUE: Nitrogen use efficiency
RCBD: Randomized complete block design

## References

[1] Liu H, Wang Z, Yu R, Li F, Li K, Cao H, Yang N, Li M, Dai J, Zan Y, Qiang Li Q, Xue C, He G, Huang D, Huang M, Liu J, Qiu W, Zhao H, Mao H (2016). Optimal nitrogen input for higher efficiency and lower environmental impacts of winter wheat production in China. Agr. Ecosyst. Environ..

[2] Guang-hao L, Gui-gen C, Wei-ping L, Da-lei L (2021). Differences of yield and nitrogen use efficiency under different applications of slow-release fertilizer in spring maize. J. Integr. Agric..

[3] Kumar VV (2018). Role of Rhizospheric Microbes in Soil.

[4] Ullah A, Noor S, Shah M, Naz R, Mahar A, ShahmirAli SK (2015). Factors affecting the adoption of organic farming in Peshawar-Pakistan. Agric. Sci..

[5] Cui Z, Zhang H, Chen X, Zhang C, Ma W, Huang C, Zhang W, Mi G, Miao Y, Li X, Gao Q, Yang J, Wang Z, Ye Y, Guo S, Lu J, Huang J, Lv S, Sun Y, Liu Y, Peng X, Ren J, Li S, Deng X, Shi X, Zhang Q, Yang Z, Tang L, Wei C, Jia L, Zhang J, He M, Tong Y, Tang Q, Zhong X, Liu Z, Cao N, Kou C, Ying H, Yin Y, Jiao X, Zhang Q, Fan M, Jiang R, Zhang F, Dou Z (2018). Pursuing sustainable productivity with millions of smallholder farmers. Nature.

[6] Zhang X, Davidson EA, Mauzerall DL, Searchinger TD, Dumas P, Shen Y (2015). Managing nitrogen for sustainable development. Nature.

[7] Alzaidi AA, Baig MB, Elhag EA (2013). An investigation into the farmers ’ attitudes towards organic farming in Riyadh Region–Kingdom of Saudi Arabia. Bulg. J. Agric. Sci..

[8] Zhihui W, Jianbo S, Blackwell M, Haigang L, Bingqiang Z, Huimin Y (2016). Combined applications of nitrogen and phosphorus fertilizers with manure increase maize yield and nutrient uptake via stimulating root growth in a long-term experiment. Pedosphere.

[9] Guang-hao L, Gui-gen C, Wei-ping L, Da-lei L (2020). Differences of yield and nitrogen use efficiency under different applications of slow release fertilizer in spring maize. J. Integr. Agric..

[10] Zant, W. Is organic fertilizer going to be helpful in bringing a green revolution to sub-Saharan Africa? Economic explorations for Malawi agriculture (Working Paper). International House Hold Survey Network (2010).

[11] Barman M, Paul S, Choudhury AG, Roy P, Sen J (2017). Biofertilizer as prospective input for sustainable agriculture in India. Int. J. Curr. Microbiol. App. Sci..

[12] Kalhapure AH, Shete BT, Dhonde MB (2013). Integrated nutrient management in maize (*Zea Mays* L.) for increasing production with sustainability. Int. J. Agric. Food Sci. Technol..

[13] Nazli RI, Kuşvuran A, Inal I, Demirbaş A, Tansi V (2014). Effects of different organic materials on forage yield and quality of silage maize (*Zea mays* L.). Turk. J. Agric. For..

[14] Niu Z, Zhang Y, Li T, Baležentis T, Štreimikienė D, Zhiyang SZ (2021). Total factor productivity growth in china’s corn farming: an application of generalized productivity indicator. J. Bus. Econ. Manag..

[15] van Wesenbeeck CFA, Keyzer MA, van Veen WCM, Qiu H (2021). Can China’s overuse of fertilizer be reduced without threatening food security and farm incomes?. Agric. Syst..

[16] Ji Y, Liu H, Shi Y (2020). Will China’s fertilizer use continue to decline? Evidence from LMDI analysis based on crops, regions and fertilizer types. PLoS ONE.

[17] Jiao X, Lyu Y, Wu X, Li H, Cheng L, Zhang C, Yuan L, Jiang R, Jiang B, Renge Z, Zhang F, Davies WJ, Shen J (2016). Grain production versus resource and environmental costs: towards increasing sustainability of nutrient use in China. J. Exp. Bot..

[18] Sher A, Khan A, Cai LJ, Ahmad MI, Asharf U, Jamoro SA (2017). Response of maize grown under high plant density; performance, issues and management: a critical review. Adv. Crop Sci. Technol..

[19] De-yang SHI, Yan-hong LI, Ji-wang Z, Peng L, Bin Z, Shu-ting D (2016). Increased plant density and reduced N rate lead to more grain yield and higher resource utilization in summer maize. J. Integr. Agric..

[20] Du X, Wang Z, Lei W, Kong L (2021). Increased planting density combined with reduced nitrogen rate to achieve high yield in maize. Sci. Rep..

[21] Li, T., Zhang, W., Yin, J., Chadwick, D., Norse, D., Lu, Y., Liu, X., Chen, X., Zhang, F., Powlson, D., & Dou, Z. Enhanced-efficiency fertilizers are not a panacea for resolving the nitrogen problem (2017). 10.1111/gcb.13918 28973790

[22] Adu-gyamfi R, Agyin-birikorang S, Tindjina I, Ahmed SM, Twumasi AD, Avornyo VK, Singh U (2019). One-time fertilizer briquettes application for maize production in savanna agroecologies of Ghana. Soil Fertil. Crop Prod..

[23] Jiang C, Ren X, Wang H (2019). Optimal nitrogen application rates of one-time root zone fertilization and the effect of reducing nitrogen application on summer maize. Sustainability.

[24] Jiang C, Lu D, Zu C, Jia Shen J, Wang S, Guo Z, Zhou J, Wang H (2018). One-time root-zone N fertilization increases maize yield, NUE and reduces soil N losses in lime concretion black soil. Sci. Rep..

[25] Li G, Zhao B, Dong S, Liu P, Vyn TJ (2017). Impact of controlled release urea on maize yield and nitrogen use efficiency under different water conditions. PLoS ONE.

[26] Sikora J, Niemiec M, Tabak M, Gródek-Szostak Z, Szelag-Sikora A, Kubon M, Komorowska M (2020). Assessment of the efficiency of nitrogen slow-release fertilizers in integrated production of carrot depending on fertilization strategy. Sustainability (Switzerland).

[27] Tian C, Zhou X, Liu Q, Peng J, Wang W, Zhan Z, Yang Y, Song H, Guan C (2016). Effects of a controlled-release fertilizer on yield, nutrient uptake, and fertilizer usage efficiency in early ripening rapeseed (*Brassica napus* L.). J. Zhejian Univ. Sci. B (Biomed. Biotechnol.).

[28] Tong D, Xu R (2012). Effects of urea and ( NH_4_)_2_SO_4_ on nitrification and acidification of Ultisols from Southern China. J. Environ. Sci..

[29] El-rokiek KG, Ahmed SA, Abd-elsamad EEH (2010). Effect of adding urea or ammonium sulphate on some herbicides efficiency in controlling weeds in onion plants. J. Am. Sci..

[30] FAO. Guidelines for soil description. Enhanced Recovery After Surgery, (2006).

[31] Landon, J. Booker Tropical Soil manual: A Handbook for Soil Survey and Agriculture Land Evaluation in the Tropics and Subtropics (2013).

[32] Zhao RF, Chen XP, Zhang FS, Zhang H, Schroder J, Romheld V (2006). Fertilization and nitrogen balance in a wheat-maize rotation system in North China. Agron. J..

[33] Huang S, Yang W, Ding W, Jia L, Jiang L, Liu Y, Xu X, Yang Y, He P, Yang J (2021). Estimation of nitrogen supply for summer maize production through a long-term field trial in china. Agronomy.

[34] Dong YJ, He MR, Wang ZL, Chen WF, Hou J, Qiu XK, Zhang JW (2016). Effects of new coated release fertilizer on the growth of maize. J. Soil Sci. Plant Nutr..

[35] Ngosong C, Bongkisheri V, Tanyi CB, Nanganoa LT, Tening AS (2019). Optimizing nitrogen fertilization regimes for sustainable maize (*Zea mays* L.) production on the volcanic soils of Buea Cameroon. Adv. Agric..

[36] Su W, Ahmad S, Ahmad I, Han Q (2020). Nitrogen fertilization affects maize grain yield through regulating nitrogen uptake, radiation and water use efficiency, photosynthesis and root distribution. PeerJ.

[37] Sainju, U. M, Ghimire, R., & Pradhan, G.P. Nitrogen Fertilization I: Impact on Crop, Soil, and Environment. IntechOpen 10.5772/intechopen.86028 (2020).

[38] Sha Z, Ma X, Wang J, Lva T, Lia Q, Misselbrook T, Liu X (2019). Effect of N stabilizers on fertilizer-N fate in the soil-crop system: a meta- analysis. Agr. Ecosyst. Environ..

[39] Chen K, Vyn TJ (2017). Post-silking factor consequences for N efficiency changes over 38 years of commercial maize hybrids. Front. Plant Sci..

[40] Jia XP, Huang JK, Xiang C, Hou LK, Zhang F, Chen XP, Cui ZL, Bergmann H (2013). Farmer’s adoption of improved nitrogen management strategies in maize production in China: an experimental knowledge training. J. Integr. Agric..

[41] Amanullah (2016). Rate and timing of nitrogen application influence partial factor productivity and agronomic NUE of maize (*Zea mays* L.) planted at low and high densities on calcareous soil in northwest Pakistan. J. Plant Nutr..

[42] Draman, A., Almas, L. K. Partial factor productivity, agronomic efficiency, and economic analyses of maize in wheat-maize cropping system in Pakistan. Southern Agricultural Economics Association Annual Meetings, 2009 (January 2009).

[43] Yan P, Zhang Q, Shuai XF, Shuai F, Pa JX, Zhang WJ, Shi JF, Wang M, Chen XP, Cui ZL (2016). Interaction between plant density and nitrogen management strategy in improving maize grain yield and nitrogen use efficiency on the North China Plain. Agric. Sci..

[44] Oenema, O. Nitrogen use efficiency (NUE) an indicator for the utilization of nitrogen in food systems. EU Nitrogen Expert Panel, January 2017, 1–4 (2015).

[45] Venterea RT, Coulter JA, Dolan MS (2016). Evaluation of intensive “4R” strategies for decreasing nitrous oxide emissions and nitrogen surplus in rainfed corn. J. Environ. Qual..

[46] Zhang C, Ju X, Powlson D, Oenema O, Smith P (2019). Nitrogen surplus benchmarks for controlling N pollution in the main cropping systems of China. Environ. Sci. Technol..

[47] Fernández C, Koop G, Steel MFJ (2013). Multiple-output production with undesirable outputs multiple-output production with undesirable outputs : an application to nitrogen surplus in agriculture. J. Am. Stat. Assoc..

[48] Børsting CF, Kristensen T, Misciattelli L, Hvelplund T, Weisbjerg MR (2003). Reducing nitrogen surplus from dairy farms. Effects of feeding and management. Livest. Prod. Sci..

[49] Liang K, Zhong X, Pan J, Huanga N, Liua Y, Penga B, Fua Y, Hu X (2019). Reducing nitrogen surplus and environmental losses by optimized nitrogen and water management in double rice cropping system of South China. Agric. Ecosyst. Environ..

[50] Klages S, Heidecke C, Osterburg B, Bailey J, Calciu I, Casey C, Dalgaard T, Frick H, Glavan M, D’Haene K, Hofman G, Leitão IA, Surdyk N, Verloop K, Velthof G (2020). Nitrogen surplus-a unified indicator for water pollution in Europe?. Water (Switzerland).

[51] Muratoglu A (2020). Grey water footprint of agricultural production: an assessment based on nitrogen surplus and high-resolution leaching runoff fractions in Turkey. Sci. Total Environ..

[52] Niemiec M, Komorowska M (2018). The use of slow-release fertilizers as a part of optimization of celeriac production technology. Agric. Eng..

[53] Ranum P, Peña-Rosas JP, Garcia-Casal MN (2014). Global maize production, utilization, and consumption. Ann. N. Y. Acad. Sci..

[54] HLPE. Biofules and food security. High Level Panel of Experts on Food Security and Nutrition of the Committee on World Food Security, Rome (2013).

[55] Karp A, Beale MH, Beaudoin F, Eastmond PJ (2015). Growing innovations for the bioeconomy. Nat. Plants.

[56] Chavarria, H., Trigo, E., Villarreal, F., Elverdin, P., & Piñeiro, V. Policy brief bioeconomy: a sustainable development strategy task force 10 sustainable energy, water, and food systems. T20, Saudi Arabia (2020).

